# Genotypes of *Staphylococcus aureus* Clinical Isolates Are Associated with Phenol-Soluble Modulin (PSM) Production

**DOI:** 10.3390/toxins14080556

**Published:** 2022-08-15

**Authors:** Harshad Lade, Sung Hee Chung, Yeonhee Lee, Hwang-Soo Joo, Jae-Seok Kim

**Affiliations:** 1 Department of Laboratory Medicine, Kangdong Sacred Heart Hospital, Hallym University College of Medicine, Seoul 05355, Korea; 2 Department of Biotechnology, College of Engineering, Duksung Women’s University, Seoul 01369, Korea

**Keywords:** *Staphylococcus aureus*, MRSA, MSSA, mass spectrometry, PSMs, delta-toxin, spa type, SCC*mec* type

## Abstract

Phenol-soluble modulins (PSMs) are important *S. aureus* virulence factors that cause cytolysis, mast cell degranulation, and stimulate inflammatory responses. In this study, PSM production by *S. aureus* clinical isolates was measured by liquid chromatography/mass spectrometry (LC-MS) and correlated with staphylococcal protein A (*spa*) type and staphylococcal cassette chromosome *mec* (SCC*mec*) type. Of 106 *S. aureus* clinical isolates, 50 (47.2%) corresponded to methicillin-susceptible *S. aureus* (MSSA) and 56 (52.8%) to methicillin-resistant *S. aureus* (MRSA). LC-MS analysis revealed no significant difference in average PSMα3, PSMα4, PSMβ2, and δ-toxin production between MSSA and MRSA isolates, but PSMα1, PSMα2, and PSMβ1 production were higher in MSSA than MRSA. This study demonstrated that average PSMα1–α4, PSMβ1–β2, and δ-toxin production by SCC*mec* type II strains was significantly lower than the IV, IVA, and V strains. Most of the SCC*mec* type II strains (*n* = 17/25; 68.0%) did not produce δ-toxin, suggesting a dysfunctional Agr system. The *spa* type t111 (except one strain) and t2460 (except one strain producing PSM α1–α4) did not produce PSMα1–α4 and δ-toxin, while average PSM production was higher among the t126 and t1784 strains. This study showed that the genotype of *S. aureus*, specifically the *spa* and SCC*mec* types, is important in characterizing the production of PSMs.

## 1. Introduction

The Gram-positive bacterium *Staphylococcus aureus* (*S. aureus*) is one of the most frequent causes of both hospital-associated (HA) and community-associated (CA) infections [1,2]. *S. aureus* infections range from skin and soft tissue infections to severe invasive diseases such as osteomyelitis, pneumonia, and bacteremia [3]. The success of *S. aureus* in the human host can be attributed to its ability to produce a wide variety of virulence factors that damage the host and evade immunity [4]. *S. aureus* can produce different virulence factors, such as enterotoxins, alpha toxins, toxic shock syndrome toxins, and phenol-soluble modulins (PSMs) [5]. PSMs, which include δ-toxin, are among the most abundant peptides in an overnight culture and can account for 60% of the total protein produced. The PSM peptide family plays a key role in *S. aureus* pathogenesis [6,7]. PSM production is regulated by the accessory gene regulator (Agr) quorum-sensing system [8,9,10], which involves the direct binding of response regulator AgrA to *psm* operon promoters [11].

PSMs are a group of amphipathic α-helical peptides that include PSMα1–PSMα4 (~20–25 amino acids), PSMβ1 and PSMβ2 (~43–45 amino acids), and δ-toxin (~26 amino acids) [6,12,13,14]. PSMs have distinct virulence functions and lyse a variety of eukaryotic cells, including neutrophils, and stimulate inflammatory responses [14,15]. PSMs also contribute to *S. aureus* pathogenicity in skin and bloodstream infections [7,16]. *Compared to other PSMs, δ-toxin* is usually a more *strongly produced peptide* that is more cytolytic to neutrophils and has a moderate capacity to stimulate formyl-peptide receptor 2 (FPR2) [6,17]. Furthermore, PSMs form bacterial functional amyloids [18] that are believed to contribute to biofilm structuring, detachment, and the systemic dissemination of biofilm-associated infection [19,20].

Members of the PSM peptide family are secreted as their primary *N*-formylated translation products by dedicated ABC exporter systems [21]. PSMs are encoded at three different locations in the *S. aureus* genome [6]. PSMα1–α4 are encoded by the *psmα* operon, PSMβ1–β2 are encoded by the *psmβ* operon, and δ-toxin is encoded by the *hld* loci. The *hld* gene is embedded within RNAIII, the RNA effector molecule of the Agr system [14]. Due to the well-known association between the Agr system and δ-toxin production, detection of δ-toxin can be used as evidence of a functional Agr system [22,23]. An additional PSM peptide, PSM-*mec,* is encoded by specific SCC*mec elements (types II, III)* of methicillin-resistant *S. aureus* (MRSA) *and is also regulated by Agr* [24,25]. Although *psm* genes are present in all *S. aureus* genomes sequenced, their expression may differ significantly [6,26]. The increased virulence of the USA300/USA500 sublineage has been attributed to the differential expression of core genome-encoded PSMs [26]. A recent study characterized variation in δ-toxin production across *S. aureus* strains and identified genetic loci associated with differences between strains [27]; however, there is little information on PSMα1–α4 and PSMβ1–β2 production by *S. aureus* strains and its association with genotypes.

An association has been reported between PSM production by MRSA and MSSA isolates in vitro and their clinical source of isolation, i.e., skin and soft tissue infection (SSTI), hospital-acquired pneumonia (HAP), and infective endocarditis (IE) [28,29]. MRSA and MSSA isolate from patients with SSTI produced higher levels of PSMα1–α4, PSMβ1, and δ-toxin than HAP or IE isolates [28,29]. However, the impact of *S. aureus* genotype differences (e.g., *S. aureus* protein A (*spa*) type and SCC*mec* type) on PSM production is largely unknown. Here, irrespective of isolates from multiple infection sites, we performed high-throughput liquid chromatography/mass spectrometry (LC-MS) analysis of 106 *S. aureus* blood culture isolates to measure in vitro PSM production. We then correlated the PSM production with *S. aureus* genotypes, specifically the *spa* and SCC*mec* types. Moreover, we focused on the clonal lineages or SCC*mec* types of MRSA and their association with PSM production.

## 2. Results

### 2.1. Molecular Characteristics of S. aureus Isolates

Among the 106 *S. aureus* isolates, 50 (47.2%) were phenotypically MSSA and 56 (52.8%) were MRSA. The *spa* typing discriminated *S. aureus* isolates into 47 types, with t189, t2460, t008, t126, t324, and t1784 as the most prevalent (Table 1). MSSA strains were distributed among 31 *spa* types, with MRSA in 21 *spa* types. SCC*mec* II (*n* = 25) and IV (*n* = 18) strains were the most predominant, followed by type IVA (*n* = 10) and V (*n* = 3).

### 2.2. In Vitro δ-Toxin Production by S. aureus Clinical Isolates

δ-toxin production is considered to be a surrogate marker of the functional Agr system in *S. aureus* [23] and dysfunction of Agr was defined as the absence of δ-toxin production [22]. In this study, δ-toxin production by *S. aureus* blood culture isolates was measured by LC-MS. We found δ-toxin production (sum of the formylated and deformylated δ-toxin) in 80 (75.5%) *S. aureus* isolates, of which 43 (86.0%) were MSSA and 37 (66.1%) were MRSA isolates (Tables S1 and S2). Of these, three MSSA and five MRSA SCC*mec* type IV strains were δ-toxin allelic variants. All δ-toxin-deficient MRSA (*n* = 19/56, 33.9%) belonged to SCC*mec* type II (*n* = 17/25, 68.0%) and type IVA (*n* = 2/10, 20.0%).

### 2.3. Association between In Vitro PSM Production, Methicillin-Resistance, SCCmec Type, and spa Type

**Figure 1:**
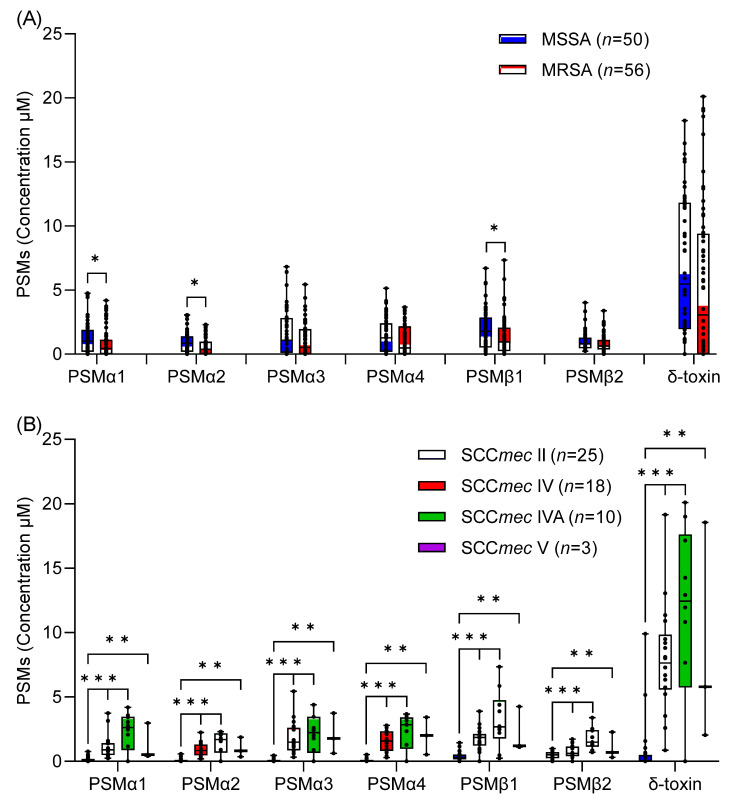
Association between PSM production and methicillin-resistance or SCC*mec* type of MRSA. (**A**) Variation in PSM production between MSSA and MRSA strains and (**B**) variation in PSM production between SCC*mec* types (II, IV, IVA, and V) of MRSA. PSM production in the culture supernatants of *S. aureus* isolates grown in TSB for 20 h at 37 °C with 200 rpm shaking was measured by LC-MS and shown as the sum of formylated and deformylated forms. Each isolate was assayed two times, and the results were averaged for each isolate and PSM. Each symbol represents one strain while the horizontal bars represent the median. Statistical analysis for the difference in average PSM production between MSSA and MRSA as well as between SCC*mec* types of MRSA was performed using separate Kruskal–Wallis tests. * *p <* 0.05, ** *p <* 0.01, *** *p <* 0.001.

The extent of PSM production in vitro was measured by LC-MS in each of the *S. aureus* blood culture isolates of different methicillin-resistance phenotypes, SCC*mec* type, and *spa* type. Within our set of isolates, no significant difference was found in average PSMα3 (1.69 μM vs. 1.11 μM), PSMα4 (1.46 μM vs. 1.05 μM), PSMβ2 (1.01 μM vs. 0.82 μM), and δ-toxin (6.81 μM vs. 5.39 μM) production between MSSA (*n* = 50) and MRSA (*n* = 56) strains by Kruskal–Wallis test, *p* > 0.05 (Figure 1A; Table S3). However, PSMα1 (1.22 μM vs. 0.88 μM), PSMα2 (0.92 μM vs. 0.63 μM), and PSMβ1 (1.92 μM vs. 1.43 μM) production were higher in MSSA than MRSA isolates. We did not compare the PSM-*mec* production of *S. aureus* isolates because its gene is located on the methicillin-resistance cassette, which MSSA lacks.

When average PSM production was compared across SCC*mec* type strains of MRSA using the separate Kruskal–Wallis test, SCC*mec* type IV (*n* = 18), IVA (*n* = 10), and V (*n* = 3) had significantly higher levels of PSMα1–α4, PSMβ1–β2, and δ-toxin production than SCC*mec* type II (*n* = 25) (*p* < 0.01) (Figure 1B; Table S4), despite each of these SCC*mec* types comprising a wide variety of *spa* types. Within SCC*mec* type II strains, *spa* type t111 (*n* = 3) and t2460 (*n* = 10) did not produce PSMα1-α4 and δ-toxin, except one t2460 strain produced lower amounts of PSMα1 (0.33 μM), PSMα2 (0.13 μM), PSMα3 (0.13 μM), and PSMα4 (0.13 μM). Alternatively, *spa* type t002 (*n* = 3) and t9353 (*n* = 3) of SCC*mec* type II produced intermediate quantities of all PSMs.

**Figure 2:**
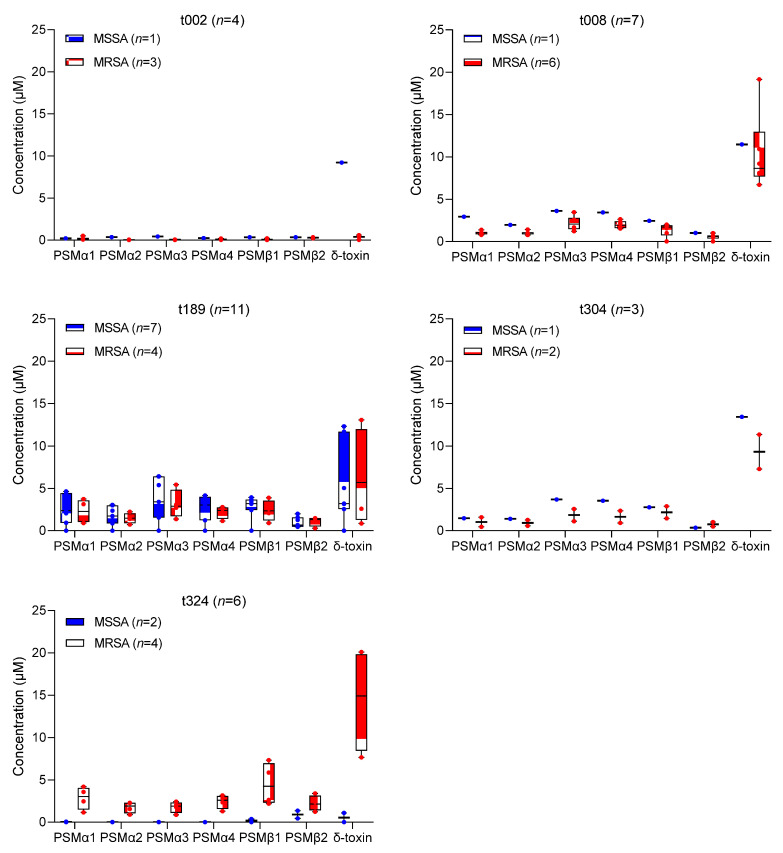
Variation in PSM production between similar *spa* types (t002, t008, t189, t304, and t324) of MSSA and MRSA strains. Difference in the average production of each PSM between MSSA and MRSA isolates was calculated using separate Kruskal–Wallis tests. No significant difference in the production of any of the PSMs was found for similar *spa* types of MSSA and MRSA (*p* > 0.05).

When variation between similar *spa* types of MSSA and MRSA t002 (1 MSSA and 3 MRSA), t008 (1 MSSA and 6 MRSA), t189 (7 MSSA and 4 MRSA), t304 (1 MSSA and 2 MRSA), and t324 (2 MSSA and 4 MRSA) was analyzed using the separate Kruskal–Wallis test, no significant difference in average PSM production was found (*p* > 0.05) (Figure 2; Table S5). However, the number of similar *spa*-type strains in each phenotypic group may be too small to reliably establish an association with PSM production.

**Figure 3:**
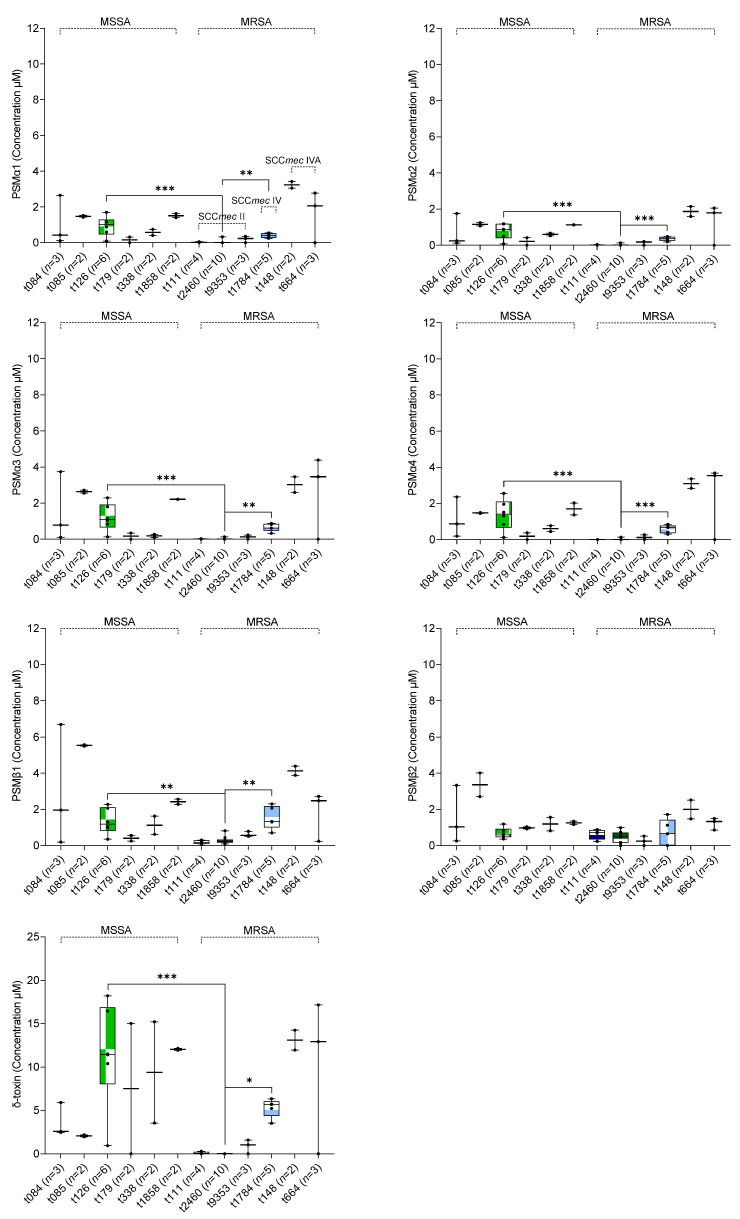
PSM production by different *spa* types (>2 strains per *spa type*) of *S. aureus*. Singleton *spa* types were not included in the figure. Difference in the average PSM production between different *spa* types was calculated using pairwise Kruskal–Wallis tests. * *p <* 0.05, ** *p <* 0.01, and *** *p <* 0.001.

When variation between the remaining different *spa* types of *S. aureus* irrespective of methicillin-resistance was analyzed using the pairwise Kruskal–Wallis test, t126 (*n* = 6) and t1784 (*n* = 5) were found to have significantly higher levels of PSMα1–α4, PSMβ1, and δ-toxin production than t2460 (*n* = 10, all δ-toxin deficient) (*p* < 0.05) (Figure 3; Table S6). Furthermore, three *spa* type t111 strains did not produce PSMα1–α4 and δ-toxin. Interestingly, all *spa* type t111 and t2460 strains showed a small amount of PSMβ1–β2 production (Figure 3). However, no significant difference in PSMβ2 production across different *spa* types of MSSA and MRSA isolates was observed (*p* > 0.05) (Figure 3). An association between the remaining *spa* types (other than t126, t1784, and t2460) and the production of PSMs could not be established due to the limited number of strains for each *spa* type.

## 3. Discussion

This study measured PSM production in *S. aureus* clinical isolates in vitro and revealed its association with strain genotypes, specifically the *spa* type and SCC*mec* type. The prevalence and molecular characteristics of the *S. aureus* isolates obtained from blood cultures of patients in a hospital showed that *spa* types t189 and t126 were dominant among MSSA isolates, while t2460, t008, and t1784 were dominant among MRSA isolates. Most of the MRSA isolates belonged to SCC*mec* type II (*n* = 25/56, 44.6%) and IV (*n* = 18/56, 32.1%). The predominance of MRSA isolates with these SCC*mec* types in this study is consistent with a previous study in Korea showing the abundance of SCC*mec* type II (*n* = 282/407, 69.2%) and IV (*n* = 97/407, 23.8%) strains [30]. Several studies have characterized *S. aureus* isolates from individual hospitals and found certain genotype strains that appear to be well adapted to the hospital environment as most prevalent [31,32,33,34,35].

The Agr system is of major importance in staphylococcal pathogenesis due to its role in the regulation of PSM production [8,9,10,14], and lack of hemolysin production therefore generally represents a dysfunctional Agr [36]. In this study, 43 (86.0%) MSSA and 37 (66.1%) MRSA isolates possessed a functional Agr system as demonstrated by semiquantitative measurement of δ-toxin production using LC-MS (Tables S1 and S2). Strikingly, most of the δ-toxin deficient MRSA strains were SCC*mec* type II (*n* = 17/19, 89.4%), with the *spa* type t2460 strains (*n* = 10/17, 58.8%) as predominant. We found that SCC*mec* type II was a significant negative predictor of δ-toxin production and thereby dysfunctional Agr system. A previous study measured δ-hemolysin activity to determine the Agr functionality and showed that SCC*mec* type II isolates which are prevalent in hospital-acquired infections in Korea are mostly Agr dysfunctional (*n* = 274/282, 90.7%) [37]. The *agrA* or *agrC* mutations were known as the main cause of Agr dysfunction in *S. aureus* clinical isolates [36,37,38,39]. Among MRSA isolates, Agr dysfunction is associated with a trend toward persistent bacteremia [37]. Furthermore, we found five SCC*mec* type IV strains of *spa* type t1784 as δ-toxin allelic variants. The amino acid sequences of δ-toxin and its allelic variant are the same with the exception at 10 (G10S) [40], due to which their molecular weights are different and can be distinguished by mass spectrometry. δ-Toxin allelic variants with highly similar peptide sequences are often present in many staphylococcal species [41].

Limited information is available about *S. aureus* PSM production and its association with specific genotypes [28,29]. We found that δ-toxin was the most strongly produced peptide, whereas low to moderate levels of PSMα1–α4 and PSMβ1–β2 were produced by *S. aureus* clinical isolates under in vitro conditions (Figure 1A). PSMs are known to be produced by all *S. aureus* strains (except naturally occurring *agr* mutants) due to the location of encoding genes on the core genome or pathogenicity islands [42], but the expression pattern may differ among them [14]. In our *S. aureus* blood culture isolates, no significant difference was observed in average PSMα3, PSMα4, PSMβ2, and δ-toxin production between MSSA and MRSA strains, but MSSA strains produced statistically higher levels of PSMα1 (*p <* 0.04), PSMα2 (*p <* 0.03), and PSMβ1 than MRSA (*p <* 0.05). Previous studies showed that the level of PSM production in *S. aureus* correlates more closely with isolation source, i.e., with the patient disease rather than with methicillin-resistance [28,29]. Another study showed that MSSA strains had significantly higher δ-toxin production than MRSA strains [27]. This difference could be due to either variation in the number of MSSA (*n* = 86) and MRSA (*n* = 38) isolates, genotypic background (Agr type and CC), or the continent of origin [27]. Indeed, we found extremely low levels of PSMα1–α4, PSMβ1–β2, and δ-toxin across SCC*mec* type II strains with nearly all *spa* type t111 (*n* = 3) and t2460 (*n* = 10) did not produce PSMα1–α4 and δ-toxin (except one t2460 strain produced low PSMα1–α4), whereas t002 and t9353 (each *n* = 3) strains of the same SCC*mec* type also produced low quantities of all PSMs.

PSM production is mainly controlled by the Agr system and strains with genetic changes leading to impaired Agr activity may be associated with decreased PSM production. A slipped-mispairing mutation in *agrA* of *S. aureus* clinical isolates results in delayed activation of Agr and failure to translate α- and δ-hemolysins [36]. The dysfunction of Agr was common among MRSA SCC*mec* type II bloodstream isolates in Korea [37]. Moreover, the transcription and translation products of *PSM-mec,* which is encoded by mobile genetic elements in SCC*mec type* II and III hospital-acquired MRSA strains have been reported to suppress the production of PSMα1–α4 [43,44]. We did not detect significant differences in average PSM production between SCC*mec* type IV, IVA, and V strains. Finally, we observed differences in average PSM production between *spa* types, with t126 and t1784 strains producing significantly higher levels of PSMα1–α4, PSMβ1, and δ-toxin than other *spa* types.

Our study has some limitations. First, *S. aureus* isolates obtained from blood cultures were only included in this study and not from multiple infection sites including SSTI, pneumonia, and surgical site infections; however, PSMs are known to be produced by all *S. aureus* strains except *agr* mutants [42]. Second, PSM production was measured in vitro, but the production of these peptides by the same *S. aureus* isolates in clinical infection may vary. Third, the significant number of singleton *spa* type strains in this study prevented meaningful associations between some *spa* types and PSM production; however, specific regions or even individual hospitals may often show a small number of particular genotype strains. Fourth, while our results do identify an association between average PSM production in vitro and some *S. aureus* genotypes (SCC*mec* type II, *spa* type t2460, t126), we do not establish the molecular basis for these associations; however, *agrA* or *agrC* mutations are known as the main cause of Agr dysfunction [36,37,38,39], which thereby suppress the PSM production including δ-toxin. Despite these limitations, our study provided quantitative data on PSM production by certain *S. aureus* genotypes, specifically *spa* type t111 and t2460 and SCC*mec* type II that would suggest low or deficient PSM production by other clinical isolates of the same genotypes, and this may inform anti-virulence strategies to treat infections caused by these genotype strains.

## 4. Conclusions

Mass spectrometric quantification showed that the production of PSMs can vary greatly depending on certain *spa* types and SCC*mec* types of *S. aureus* isolates. Our results suggest that *S. aureus* SCC*mec* type II strains with *spa* type t111 and t2460 are associated with deficient PSMα1–α4 and δ-toxin production, except for one strain of each *spa*. Further, *S. aureus spa* type t126 and t1784 strains are associated with higher levels of PSM production than other *spa* types. Indeed, this study quantitatively measured PSM production of *S. aureus* clinical isolates and revealed that it was associated with certain genotype strains.

## 5. Materials and Methods

### 5.1. Bacterial Strains and Growth Conditions

A total of 106 *S. aureus* isolates obtained from blood cultures of patients at Kangdong Sacred Heart Hospital, Seoul, Korea from 2020 to 2021 were included in this study. All the isolates were identified by the matrix-assisted laser desorption ionization/time-of-flight mass spectrometry (MALDI-TOF MS) system (Bruker Microflex LT, Bruker Daltonik GmbH, Bremen, Germany). The presence of a gene encoding methicillin-resistance was confirmed by polymerase chain reaction (PCR) screening for *mecA* gene [45]. Stock cultures of isolates were stored frozen (−70 °C) in skimmed milk.

### 5.2. The spa Typing and SCCmec Typing

The *spa* typing of *S. aureus* isolates was performed as described previously [46]. A single locus of the repeat region X of the *spa* gene was sequenced and analyzed using Ridom StaphType software (http://spaserver.ridom.de/, accessed on 13 September 2021). The *spa* types were assigned using the BioNumerics software v.7.5 (Applied Math, Sint-Martens-Latem, Belgium). All MRSA strains were subjected to SCC*mec* typing by discriminating the *mec* gene complex and the cassette chromosome recombinases (*ccr*) gene complex types [47].

### 5.3. PSM Quantification by LC–MS

Quantification of PSM production in the culture supernatants was performed using LC-MS as described previously [48]. Briefly, *S. aureus* strains were grown in 200 μL of TSB in a 96-well microtiter plate (Corning 3596, NY) for 20 h at 37 °C with 200 rpm shaking. Cultures were pelleted at 3100× *g* for 20 min at 4 °C, and supernatants were used for PSM quantification by LC-MS.

Aliquots (5 μL) of harvested supernatants were eluted from a C8 column (ZORBAX SB-C8, 2.1 × 5 mm, 1.8 µm; Agilent, Santa Clara, CA, USA) on a Waters ZQ 2000 LC-MS system (Waters, Milford, MA) with a gradient of trifluoroacetic acid (TFA; 0.05%) in water and 0.05% TFA in acetonitrile at a 0.3 mL/min flow rate. Electrospray ionization of samples was performed at 3.5 kV and ions were infused into the ion entrance of a mass spectrometer. The *m*/*z* values of the analytes were scanned continuously, and mass spectra were recorded. The *m*/*z* values of 2+ and 3+ charged ions of α-type PSMs and 3+ and 4+ charged ions of β-type PSMs were used to extract chromatograms for quantification of each PSM. The peptides were quantified by the sum of extracted ion chromatograms of formylated and deformylated forms. The concentration of PSMs was determined by external calibration with synthetic formyl PSMs. Formyl PSM peptides were synthesized by Peptron (Daejeon, Korea) and Cosmogenetech (Daejeon, Korea). The cultivation and mass analysis were performed in duplicate for each isolate.

### 5.4. Statistical Analysis

Statistical analyses were performed with SPSS software version 24.0 (SPSS Inc., IBM, Chicago, IL, USA). PSM production by each isolate was quantified in duplicate and averaged. The PSM values were compared using the Kruskal–Wallis tests and differences in production across methicillin-resistance, SCC*mec* type, and *spa* type were considered statistically significant if * *p <* 0.05, ** *p <* 0.01, and *** *p <* 0.001. The plots were created using GraphPad Prism software version 9.3.0 (GraphPad Software Inc., San Diego, CA, USA).

## Figures and Tables

**Table 1:** Genotype distribution of *S. aureus* clinical isolates.

*spa* Type	*S. aureus* Isolates *n* (%)	Phenotype		SCC*mec* Type for MRSA
		MSSA *n* (%)	MRSA *n* (%)	
**MSSA and MRSA**				
t189	11 (10.4)	7 (6.6)	4 (3.8)	IV
t008	7 (6.6)	1 (0.9)	6 (5.7)	IV
t324	6 (5.7)	2 (1.9)	4 (3.8)	IVA
t002	4 (3.8)	1 (0.9)	3 (2.8)	II
t304	3 (2.8)	1 (0.9)	2 (1.9)	IV
**MSSA**				
t126	6 (5.7)	6 (5.7)	-	O
t084	3 (2.8)	3 (2.8)	-	O
t085	2 (1.9)	2 (1.9)	-	O
t179	2 (1.9)	2 (1.9)	-	O
t338	2 (1.9)	2 (1.9)	-	O
t1858	2 (1.9)	2 (1.9)	-	O
t005	1 (0.9)	1 (0.9)	-	O
t019	1 (0.9)	1 (0.9)	-	O
t021	1 (0.9)	1 (0.9)	-	O
t127	1 (0.9)	1 (0.9)	-	O
t177	1 (0.9)	1 (0.9)	-	O
t346	1 (0.9)	1 (0.9)	-	O
t363	1 (0.9)	1 (0.9)	-	O
t386	1 (0.9)	1 (0.9)	-	O
t416	1 (0.9)	1 (0.9)	-	O
t521	1 (0.9)	1 (0.9)	-	O
t571	1 (0.9)	1 (0.9)	-	O
t1333	1 (0.9)	1 (0.9)	-	O
t1361	1 (0.9)	1 (0.9)	-	O
t1767	1 (0.9)	1 (0.9)	-	O
t1950	1 (0.9)	1 (0.9)	-	O
t4727	1 (0.9)	1 (0.9)	-	O
t4956	1 (0.9)	1 (0.9)	-	O
t10234	1 (0.9)	1 (0.9)	-	O
t10686	1 (0.9)	1 (0.9)	-	O
t12605	1 (0.9)	1 (0.9)	-	O
undefined	1 (0.9)	1 (0.9)	-	O
**MRSA**				
t2460	10 (9.4)	-	10 (9.4)	II
t1784	5 (4.7)	-	5 (4.7)	IV
t111	4 (3.8)	-	4 (3.8)	II
t664	3 (2.8)	-	3 (2.8)	IVA
t9353	3 (2.8)	-	3 (2.8)	II
t148	2 (1.9)	-	2 (1.9)	IVA
t034	1 (0.9)	-	1 (0.9)	V
t062	1 (0.9)	-	1 (0.9)	II
t242	1 (0.9)	-	1 (0.9)	IV
t264	1 (0.9)	-	1 (0.9)	II
t893	1 (0.9)	-	1 (0.9)	II
t1081	1 (0.9)	-	1 (0.9)	V
t1154	1 (0.9)		1 (0.9)	II
t1560	1 (0.9)	-	1 (0.9)	II
t3092	1 (0.9)	-	1 (0.9)	V
t4359	1 (0.9)	-	1 (0.9)	IVA

MRSA, methicillin-resistant *S. aureus*; MSSA, methicillin-sensitive *S. aureus*; *n*, number of isolates; SCC*mec,* staphylococcal cassette chromosome *mec;* *spa*, staphylococcal protein A; -, none; O, non-typeable.

## Data Availability

The data presented in this study are available in this article and Supplementary Material.

## Supplementary Materials

The following supporting information can be downloaded at https://www.mdpi.com/article/10.3390/toxins14080556/s1: Table S1. Genotypic characteristics and δ-toxin production of MSSA clinical isolates; Table S2. Genotypic characteristics and δ-toxin production of MRSA clinical isolates; Table S3. Variation in PSM production between MSSA and MRSA strains; Table S4. Variation in PSM production between SCCmec type strains of MRSA; Table S5. Variation in PSM production between similar spa type strains of MSSA and MRSA; Table S6. PSM production by different spa type strains of S. aureus clinical isolates; Figure S1. δ-toxin deficient MSSA (n = 7) and MRSA (n = 19) strains.
