# Supplementary material for: Genotypes of Staphylococcus aureus Clinical Isolates Are Associated with Phenol-Soluble Modulin (PSM) Production
Source: Toxins (Basel). 2022 Aug 15;14(8):556. doi: 10.3390/toxins14080556 (PMC9412541; doi:10.3390/toxins14080556)
Supplement: Supplementary file 1 [file toxins-14-00556-s001.zip › toxins-1842462-supplementary.pdf]

Toxins **2022**, *14*, 556; doi: 10.33390/toxins14080556

Supplementary Materials: Genotypes of *Staphylococcus aureus* Clinical Isolates Are Associated with Phenol-Soluble Modulin (PSM) Production

Harshad Lade, Sung Hee Chung, Yeonhee Lee, Hwang-Soo Joo and Jae-Seok Kim

Table S1. Genotypic characteristics and δ-toxin production of MSSA clinical isolates.

MSSA strains	*spa* type	δ-toxin production
24689	t002	+
26136	t005	+
24865	t008	+
26782	t019	+
27126	t021	+
25139	t084	+
25496	t084	+
26486	t084	+
26559	t085	+
26641	t085	+
24692	t126	+
24899	t126	+
24900	t126	+
25157	t126	+
27043	t126	+
27254	t126	+
25280	t127	-
26768	t177	-
25180	t179	-
26704	t179	+
26052	t189	+
26253	t189	+
26255	t189	+
26331	t189	+
26369	t189	-
26537	t189	+
27339	t189	+
26340	t304	+
24855	t324	-
24901	t324	+
25992	t338	+
26653	t338	+
25637	t346	+
26287	t363	+
26642	t386	+
25499	t416	+
26931	t521	+
27078	t571	+

24915	t1333	+
26170	t1361	+
26153	t1767	+
25350	t1858	+
25351	t1858	+
26281	t1950	-
24722	t4727	-
25930	t4956	+
25106	t10234	+
26612	t10686	+
25619	t12605	+
25025	undefined	+

+, present; -, absent.

Table S2. Genotypic characteristics and δ-toxin production of MRSA clinical isolates.

MRSA strains	*spa* type	SCC*mec* type	δ-toxin production
24663	t002	II	-
26180	t002	II	+
26744	t002	II	+
24651	t008	IV	+
25250	t008	IV	+
25773	t008	IV	+
26154	t008	IV	+
26928	t008	IV	+
27310	t008	IV	+
25870	t034	V	+
26792	t062	II	+
25754	t111	II	+
26558	t111	II	-
27106	t111	II	-
27179	t111	II	-
24718	t148	IVA	+
27186	t148	IVA	+
25849	t189	IV	+
26194	t189	IV	+
26644	t189	IV	+
26930	t189	IV	+
24962	t242	IV	+
26725	t264	II	-
24958	t304	IV	+
26538	t304	IV	+
25029	t324	IVA	+
26393	t324	IVA	+
26421	t324	IVA	+
27331	t324	IVA	+
25027	t664	IVA	-
25944	t664	IVA	+
26031	t664	IVA	+

26507	t893	II	+
25274	t1081	V	+
24952	t1154	II	-
25620	t1560	II	+
24733	t1784	IV	+
25135	t1784	IV	+
26216	t1784	IV	+
26987-2	t1784	IV	+
27288	t1784	IV	+
25437	t2460	II	-
25606	t2460	II	-
25625	t2460	II	-
25681	t2460	II	-
25753	t2460	II	-
26088	t2460	II	-
26209	t2460	II	-
26256	t2460	II	-
26271	t2460	II	-
26793	t2460	II	-
24830	t3092	V	+
25580	t4359	IVA	-
25899	t9353	II	-
26101	t9353	II	+
26139	t9353	II	+

+, present; -, absent.

Table S3. Variation in PSM production between MSSA and MRSA strains.

PSM subtype	Average PSM production (μM)		*P* value
	MSSA (*n* = 50)	MRSA (*n* = 56)	
**PSMα1**	1.229	0.882	0.036
PSMα2	0.929	0.635	0.030
PSMα3	1.693	1.116	0.061
PSMα4	1.462	1.056	0.055
PSMβ1	1.925	1.434	0.048
PSMβ2	1.018	0.820	0.141
δ-toxin	6.811	5.391	0.058

Averaged PSM production by MSSA and MRSA strains. Kruskal–Wallis test was used to calculate the difference in production. There was no significant difference in PSMα3, PSMα4, PSMβ2, and δ-toxin production between MSSA and MRSA strains, but MSSA strains produce higher amounts of PSMα1, PSMα2, and PSMβ1 than MRSA, $P > 0.05$.

Table S4. Variation in PSM production between SCC*mec* type strains of MRSA.

PSM subtype	Average PSM production (μM)			
	**SCC*mec* II (*n* = 25)**	**SCC*mec* IV (*n* = 18)**	**SCC*mec* IVA (*n* = 10)**	**SCC*mec* V (*n* = 3)**
PSMα1	0.120	1.097	2.272	1.304
PSMα2	0.068	0.902	1.459	1.014
PSMα3	0.058	1.883	2.100	2.043
PSMα4	0.073	1.572	2.308	1.984
PSMβ1	0.395	1.758	3.220	2.189
PSMβ2	0.488	0.731	1.730	1.090
δ-toxin	0.785	7.891	11.390	8.790

Averaged PSM production by SCC*mec* type strains of MRSA. The SCC*mec* type IV, IVA, and V strains had significantly higher PSMα1-α4, PSMβ1-β2, and δ-toxin production than SCC*mec* type II strains.

Table S5. Variation in PSM production between similar *spa* type strains of MSSA and MRSA.

***spa* type and PSMs**	**Average PSM production (μM)**		***P* value**
***spa* type t002 (*n* = 4)**	**MSSA (*n* = 1)**	**MRSA (*n* = 3)**	
PSMα1	0.210	0.242	0.655
PSMα2	0.352	0.026	0.180
PSMα3	0.429	0.043	0.180
PSMα4	0.246	0.097	0.180
PSMβ1	0.340	0.083	0.180
PSMβ2	0.345	0.282	0.655
δ-toxin	9.211	0.331	0.180
***spa* type t008 (*n* = 7)**	**MSSA (*n* = 1)**	**MRSA (*n* = 6)**	
PSMα1	2.923	0.985	0.134
PSMα2	1.958	0.974	0.134
PSMα3	3.616	2.147	0.134
PSMα4	3.434	1.985	0.134
PSMβ1	2.440	1.374	0.134
PSMβ2	1.012	0.546	0.134
δ-toxin	11.470	10.343	0.134
***spa* type t189 (*n* = 11)**	**MSSA (*n* = 7)**	**MRSA (*n* = 4)**	
PSMα1	2.505	2.315	0.850
PSMα2	1.824	1.478	0.345
PSMα3	3.725	3.149	0.571
PSMα4	2.642	2.193	0.186
PSMβ1	2.849	2.368	0.450
PSMβ2	1.017	1.108	1.000
δ-toxin	5.427	6.328	0.850
***spa* type t304 (*n* = 3)**	**MSSA (*n* = 1)**	**MRSA (*n* = 2)**	
PSMα1	1.469	1.024	1.000
PSMα2	1.408	0.924	0.221
PSMα3	3.700	1.854	0.221
PSMα4	3.542	1.644	0.221
PSMβ1	2.771	2.174	1.000
PSMβ2	0.361	0.753	0.221
δ-toxin	13.430	9.328	0.221
***spa* type t324 (*n* = 6)**	**MSSA (*n* = 2)**	**MRSA (*n* = 4)**	
PSMα1	0.031	2.849	0.064
PSMα2	0.015	1.752	0.064

PSMα3	0.014	1.773	0.064
PSMα4	0.005	2.413	0.064
PSMβ1	0.188	4.506	0.064
PSMβ2	0.904	2.228	0.165
δ-toxin	0.544	14.400	0.064

Averaged PSM production by similar *spa* type strains of MSSA and MRSA. The separate Kruskal–Wallis test was used to calculate the difference in production. There was no significant difference in PSMα1-α4, PSMβ1-β2, and δ-toxin production between similar *spa* type strains of MSSA and MRSA, $P > 0.05$.

Table S6. PSM production by different *spa* type strains of *S. aureus* clinical isolates.

spa types	PSMα1	PSMα2	PSMα3	PSMα4	PSMβ1	PSMβ2	δ-toxin
**MSSA**							
t084 (*n* = 3)	1.065	0.707	1.547	1.139	2.950	1.542	3.658
t085 (*n* = 2)	1.476	1.159	2.635	1.473	5.540	3.363	2.077
t126 (*n* = 6)	0.932	0.779	1.207	1.381	1.338	0.675	11.489
t179 (*n* = 2)	0.159	0.210	0.172	0.184	0.402	0.979	7.515
t338 (*n* = 2)	0.584	0.598	0.188	0.607	1.125	1.192	9.383
t1858 (*n* = 2)	1.513	1.127	2.214	1.697	2.424	1.254	12.048
**MRSA**							
t111 (*n* = 4)	0.015	0.008	0.007	0.000	0.184	0.638	0.073
t2460 (*n* = 10)	0.033	0.014	0.013	0.013	0.290	0.474	0.000
t9353 (*n* = 3)	0.199	0.127	0.122	0.128	0.622	0.256	0.873
t1784 (*n* = 5)	0.403	0.364	0.653	0.583	1.541	0.701	5.305
t148 (*n* = 2)	3.240	1.868	3.028	3.101	4.138	1.998	13.108
t664 (*n* = 3)	1.615	1.283	2.617	2.408	1.811	1.227	10.028

Averaged PSM production by different *spa* type strains.

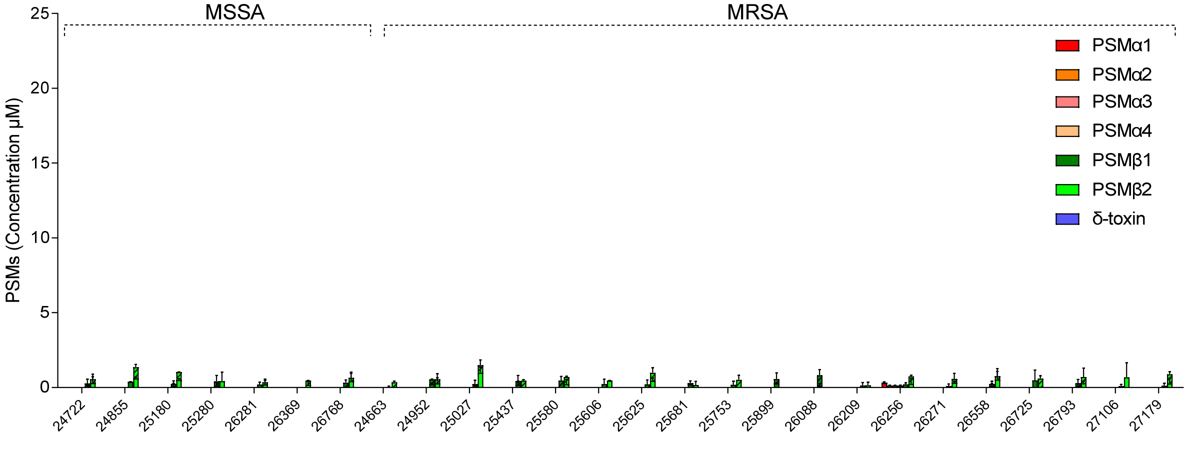

Figure S1. δ-toxin deficient MSSA (*n* = 7) and MRSA (*n* = 19) strains. PSM production was measured by LC-MS and shown as the sum of formyl- and deformylated forms. All the δ-toxin deficient strains also showed no production of PSMα1-α4, with strain 26256 as exception. Most of the δ-toxin deficient strains produced low quantity of PSMβ1-β2.
